# Associations of Immune Genetic Variability with Gulf War Illness in 1990–1991 Gulf War Veterans from the Gulf War Illness Consortium (GWIC) Multisite Case-Control Study

**DOI:** 10.3390/brainsci11111410

**Published:** 2021-10-26

**Authors:** Janet K. Coller, Jonathan Tuke, Taylor J. Wain, Emily Quinn, Lea Steele, Maria Abreu, Kristina Aenlle, Nancy Klimas, Kimberly Sullivan

**Affiliations:** 1 Discipline of Pharmacology, School of Biomedicine, University of Adelaide, Adelaide 5005, South Australia, Australia; taylor.wain@adelaide.edu.au; 2 School of Mathematical Sciences, University of Adelaide, Adelaide 5005, South Australia, Australia; simon.tuke@adelaide.edu.au; 3 Biostatistics and Epidemiology Data Analytics Center, Boston University School of Public Health, Boston, MA 02118, USA; eq@bu.edu; 4 Veterans Health Research Program, Beth K. and Stuart C. Yudofsky Division of Neuropsychiatry, Department of Psychiatry and Behavioral Sciences, Baylor College of Medicine, Houston, TX 77030, USA; Lea.Steele@bcm.edu; 5 Institute for Neuroimmune Medicine, Dr. Kiran C. Patel College of Osteopathic Medicine, Nova Southeastern University, Fort Lauderdale, FL 33314, USA; mabreu1@nova.edu (M.A.); kaenlle@nova.edu (K.A.); nklimas@nova.edu (N.K.); 6 Department of Veterans Affairs, Research Service, Miami VA Healthcare System, Miami, FL 33125, USA; 7 Department of Veterans Affairs, Miami VA Healthcare System Geriatric Research Education and Clinical Center Healthcare System, Miami, FL 33125, USA; 8 Department of Environmental Health, Boston University School of Public Health, Boston, MA 02118, USA; tty@bu.edu

**Keywords:** Gulf War illness, immune genetics, toll-like receptor 4, predictive genetic model, objective biomarker

## Abstract

Gulf War illness (GWI) encompasses a constellation of persistent debilitating symptoms associated with significant changes in central nervous system (CNS) and immune functioning. Currently, there is no validated biomarker for GWI risk susceptibility. Given the impact of immune responses linked to GWI symptomology, genetic variability that causes persistent inflammatory/immune alterations may be key. This Boston University-based Gulf War Illness Consortium (GWIC) study investigated the impact of single nucleotide polymorphisms (SNPs) in variants of immune and pain genetic markers *IL1B*, *IL2*, *IL6*, *IL6R*, *IL10*, *TNF*, *TGF*, *TLR2*, *TLR4*, *MD2*, *MYD88*, *BDNF*, *CRP*, *ICE, COMT* and *OPRM1* on GWI occurrence in a Caucasian subset of Gulf War (GW) veterans with (cases, *n* = 170) and without (controls, *n* = 34) GWI. Logistic regression modeling created a prediction model of GWI risk that associated genetic variability in *TGF* (rs1800469, *p* = 0.009), *IL6R* (rs8192284, *p* = 0.004) and *TLR4* (rs4986791, *p* = 0.013) with GWI occurrence. This prediction model was specific and sensitive, with a receiver operator characteristic area under the curve of 71.4%. This is the first report of immune genetic variability being predictive of GWI and warrants validation in larger independent cohorts. Future reports will present interactions of these genetic risk factors with other characteristics of GW service.

## 1. Introduction

Gulf War illness encompasses a constellation of persistent debilitating symptoms suffered by a third of the nearly 700,000 U.S. soldiers who served in the 1990–1991 Persian Gulf War (GW) [1]. Symptoms of GWI include persistent fatigue, cognitive difficulties and musculoskeletal pain and these symptoms are associated with significant changes in central nervous system (CNS) and immune functioning [2,3,4,5]. There is now mounting evidence demonstrating the importance of CNS inflammatory markers and immune system activation in the development of chronic symptoms in GW veterans [6]. In addition, there are studies showing lower white matter volumes and increased microstructural diffusivity on brain imaging in GW veterans exposed to neurotoxicants linked to increased health symptom complaints [3,4,7,8,9]. This suggests CNS neuro-immune signaling (glial) cells may have an important role in the development and sustained health symptom and cognitive decrements associated with GWI [10]. In particular, myelin and neuronal breakdown products in the extracellular spaces are thought to activate glial cells by acting as toll-like receptor (TLR) agonists, specifically at TLR4 [11,12]. Glial activation of TLR4 from these internal factors (including HMGB1 [13]), as well as external stimuli (e.g., cellular debris, bacteria) results in release of CNS pro-inflammatory cytokines (e.g., interleukin (IL)-1, IL-6, tumor necrosis factor (TNF)). The CNS inflammatory response [14] induces sickness response symptoms including fatigue, muscle and joint pain and cognitive difficulties. These symptoms are similar to those reported by ill GW veterans and are exacerbated with multiple stimuli such as mild traumatic brain injuries and neurotoxicant exposure [15]. Hence neuroimmune pathway activation is a likely mechanism contributing to GWI. However, what is less clear is why only some GW veterans have chronic illness while others with similar exposures do not, and may be suggestive of variability in genetic susceptibility for chronic inflammation or risk susceptibility of GWI.

Currently, there is no validated biomarker for risk susceptibility to GWI. One study has suggested that genes in the major histocompatibility class (MHC) II family of human leukocyte antigens (HLA) may be a potential risk factor [16]. Investigators have also identified significant associations between GWI and genetic variants of the enzymes butyrylcholinesterase (BChE) and paraoxonase-1 (PON1), which act to neutralize adverse effects of cholinergic neurotoxicants on the body [17,18]. Given the impact of immune responses linked to GWI symptomology and the substantial variability in immune response between individuals, we hypothesized that likely biomarkers may reside in genes that control or enhance inflammation such as TGF-beta (TGF-β, TGF). This Boston University-based Gulf War Illness Consortium (GWIC) study aimed to investigate the associations between a number of immune and pain genetic loci and GWI in GW veterans with and without GWI.

## 2. Materials and Methods

### 2.1. Study Participants

Two-hundred and sixty-nine GW veterans with (cases, *n* = 223) and without (controls, *n* = 46) GWI were recruited to participate in the core GWIC case-control study [19]. The study protocol and informed consent documents were approved by institutional review boards at Boston University, Miami VAMC, and Baylor College of Medicine and reviewed by the U.S. Army Medical Research and Development Command’s Office of Human Research Protections. All participants provided written informed consent prior to participating in accordance with the Declaration of Helsinki. Eligibility criteria and GWI case/control status determined according to the Kansas GWI case definition criteria have been described previously [19]. In particular, veterans who had previously been diagnosed with any from a predetermined list of chronic medical conditions were excluded from recruitment in the study. Data were collected on each participant’s deployment and medical history, physical characteristics (e.g., vital signs, body mass index, smoking status) and demographic characteristics including sex, age, race, and ethnicity.

### 2.2. Genetic Analysis

Genomic DNA was isolated from saliva samples from each veteran and analyzed for 21 single nucleotide polymorphisms (SNPs) in the following genes using a customized Agena Mass Array assay at the Australian Genome Research Facility (Brisbane, Australia): *IL1B* (rs16944, rs1143627, rs1143634), *IL2* (rs2069762), *IL6* (rs10499563), *IL10* (rs1800871, rs1800896), *IL6R* (rs8192284/rs2228145), *TNF* (rs1800629), *TGF* (rs11466314, rs1800469), *TLR2* (rs3804100), *TLR4* (rs4986790, rs4986791), *MD2* (LY96, rs11466004), *MYD88* (rs6853), *BDNF* (rs6265), *CRP* (rs2794521), *ICE* (CASP1, rs554344, rs580253) and *OPRM1* (rs1799971) [20]. In addition, variability in the *COMT* (rs4680) gene was examined using a commercially available TaqMan^®^ SNP genotyping assay kit (ThermoFisher Scientific, Scoresby, Australia).

### 2.3. Statistical Analysis

Hardy–Weinberg equilibrium was not significant for any SNP (*p* > 0.77). In order to avoid race obscuring associations, data from Caucasian only veterans with complete genetic information, 170 GWI cases and 34 controls, were included for analyses reported here. Similar analyses in other ethnic groups were not possible due to small sample number of cases (*n* = 2–4). Chi-square, Fisher’s exact and Mann–Whitney U tests were used to examine differences in participant demographic, deployment and military characteristics and genotypes between veterans with (cases) versus without (controls) GWI. Logistic regression modeling with step-wise approach of model building, where each genetic factor was added to investigate increased strength of the model and then once a genetic factor was added a step-back was performed to see if the model could be further improved by removing a previously added genetic factor. Factors were added or removed based on Akaike information criterion (AIC). This process built the strongest predictive genetic risk model. The receiver operator characteristic (ROC) curve was obtained by plotting the sensitivity (true positive) of the model against 1-specificity (false positive) of the model [21]. The percentage area under the curve (AUC) of the ROC curve was then used to assess the ability of the models to predict GWI with the value indicating the percentage of veterans who were correctly classified by the model as having GWI. Analysis was conducted using R [22] in RStudio [23].

## 3. Results

Participant demographic, military, and deployment characteristics are presented in Table 1. These characteristics were not different between GWI cases and controls with the exception of the 1990 rank of the veterans, where there was a significantly higher number of officers in the control group (Odds ratio (95% confidence interval) = 3.8 (1.6–8.8), *p* = 0.006, Table 1).

With regard to the individual SNPs, for both the *TGF* and *IL6R* SNPs cases had a lower frequency occurrence of the wild-type genotype (C/C or A/A) and higher frequency occurrence of the heterozygote (C/T or A/C) and homozygous variant (T/T or C/C) genotypes compared to controls (Table 2). Whilst for the *TLR4* SNP, cases had a higher frequency occurrence of the wild-type (C/C) genotype and a lower frequency occurrence of the heterozygote variant (C/T) genotype compared to controls (Table 2).

**Figure 1:**
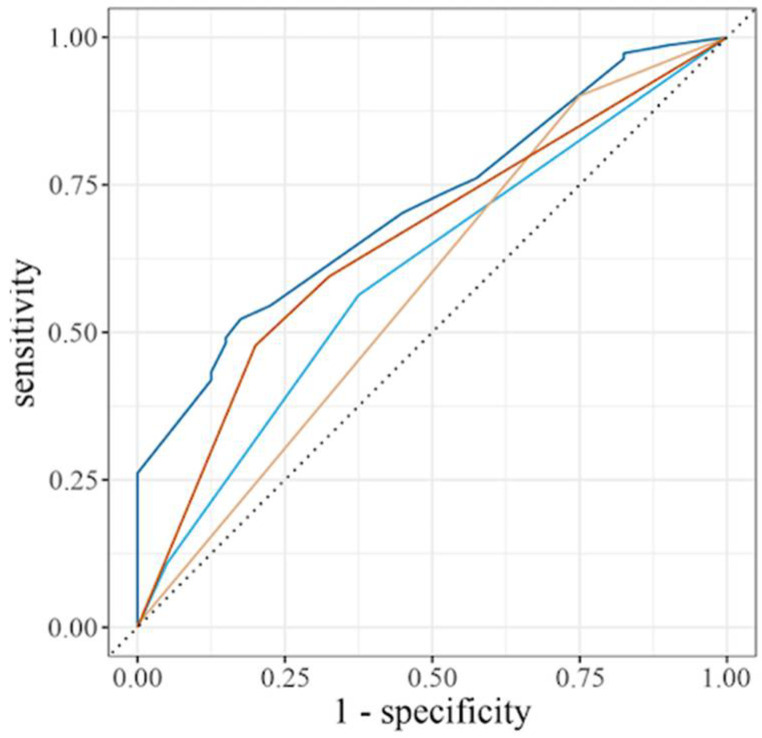
Receiver operator characteristic (ROC) curves of individual SNPs from *TGF* (rs1800469, AUC 60%, light blue line), *IL6R* (rs8192284, AUC 65%, red line), and *TLR4* (rs4986791, AUC 58%, yellow line), and all three SNPs (AUC 71%, dark blue line) to predict GWI.

The predictive GWI logistic regression model included genetic variability in *TGF* (rs1800469, *p* = 0.009), *IL6R* (rs8192284, *p* = 0.004), and *TLR4* (rs4986791, *p* = 0.013). The ROC curve AUCs for each individual SNP as well as the combination of all three SNPs were: rs4986791 58%; rs1800469 60%; rs8192284 65%; and all three SNPs 71% (Figure 1). Consequently, the model with all three SNPs was the most specific and sensitive for predicting GWI as indicated by the ROC curve with the highest AUC.

## 4. Discussion

This GWIC study is the first to model the impact of genetic variability in pro-inflammatory and anti-inflammatory cytokines and receptors, innate immune response pathways and pain signaling pathways in determining risk of GWI. The risk prediction model that included all three SNPs from *TGF* (rs1800469), *IL6R* (rs8192284), and *TLR4* (rs4986791) was the most specific and sensitive as evidenced by the highest ROC curve % AUC, with the model having a 71% chance of correctly classifying a veteran with GWI, an AUC considered in the statistical literature as being acceptable [21].

Prior to discussing the implications of this model, it is important to note that the only difference between GWI cases and controls in participant demographic, military and deployment characteristics within the subset of Caucasian GWIC veterans was the higher proportion who served in the enlisted ranks versus officers during deployment. This was reported previously for the entire GWIC cohort [19] and is similar to previous studies of GWI [24,25,26]. However, this is unlikely to impact on the overall genetic associations reported in this study.

Of the genetic variants included in the model, the *TGF* SNP resides in the promoter region of the gene and the variant has been linked to higher serum TGF-β concentrations [27]. With regard to the *IL6R* SNP, this resides in the coding region (exon 9) of the gene and the variant has been linked to higher levels of soluble IL-6 receptor [28] and therefore has the potential for increased IL-6 inflammatory signaling. Interestingly, a previous review has discussed that in the presence of both TGF-β and IL-6 signaling in inflammatory conditions, differentiation of Th17 cells occurs; this can further drive inflammation [29]. Consequently, as both of these cytokines are increased in the presence of variant SNPs in response to immune challenges, such as deployment exposure to neurotoxicants, heightened and sustained inflammatory responses are likely. This can potentially explain the higher frequency of *TGF* and *IL6R* variant genotype carriers in GWI cases compared to controls. This hypothesis is also in line with other studies that have demonstrated a link between increased IL-6 expression and other chronic inflammatory diseases such as rheumatoid arthritis [30] and inflammatory bowel disease [31].

With regard to the *TLR4* SNP, this resides in the coding region (exon 4) of the gene (1196C > T) and the variant causes an amino acid change (Thr399Ile) in TLR4 in the extracellular domain, with the variant associated with lessened response to TLR4 agonist (lipopolysaccharide) binding as indicated by reduced nuclear factor kappa B signaling [32]. That is, having a copy of this variant decreases inflammatory signaling. Consequently, this could explain why veterans with this variant are less likely to have GWI.

In conclusion, this is the first GWI genetic risk model to implicate immune genetic variability of the *TGF*, *IL6R* and *TLR4* genes being markers of risk that is both specific and sensitive. These preliminary outcomes have the potential to assist in identifying therapies to improve the daily life of GW veterans living with GWI through personalizing treatment based on genetics. However, these findings must be considered within the context of the limitations of this study, notably the small sample size of the control group and that information regarding additional deployment and health factors that have the potential to impact on risk of GWI were not collected or not included in these preliminary assessments. Validation studies would need to consider these factors and are also needed in larger independent veteran populations to further evaluate our results. In addition, as the genetic risk model generated could not predict 100% of GWI cases, improvement of the model through addition of other participant demographics and lifestyle factors (including body mass index and smoking status), and deployment characteristics is ongoing in this subset of GWIC veterans.

## Figures and Tables

**Table 1:** Demographic, military and deployment characteristics of Caucasian GWI cases and controls, data are *n* (%). *p*-values were obtained by Chi-square, Fisher’s exact or Mann–Whitney U tests comparing the two groups.

	Cases (*n* = 170) *	Controls (*n* = 34)	*p*
Sex			
Male	143 (84)	31 (91)	0.43
Female	27 (16)	3 (9)	
Age (years)			
43–49	59 (35)	10 (29)	0.56
50–59	91 (54)	17 (50)	
60–69	17 (10)	6 (18)	
70+	3 (2)	1 (3)	
Median age (years)	51	53	0.15
Highest Education level			0.93
High school or GED	8 (5)	2 (6)	
Some college or training after high school	85 (50)	15 (44)	
4 year degree	35 (21)	8 (24)	
Advanced degree	42 (25)	9 (26)	
Rank in 1990 *			0.006
Enlisted	150 (89)	23 (68)	
Officer	19 (11)	11 (32)	
Branch of Service in 1990 *			0.59
Army	114 (67)	24 (70)	
Navy	16 (9)	5 (15)	
Air Force	11 (7)	2 (6)	
Marines	28 (17)	3 (9)	
Service Component in 1990 *			0.96
Regular (Active Component)	126 (75)	26 (76)	
Reserves	31 (18)	6 (18)	
National Guard	12 (7)	2 (6)	
Gulf War Deployment: Service period in theater *			
Departed prior to Jan 1991	3 (2)	1 (3)	0.25
Present Jan-Feb 1991, departed by May 1991	119 (70)	24 (70)	
Present Jan-Feb 1991, departed after May 1991	35 (21)	5 (15)	
Arrived in March 1991 or later	12 (7)	4 (12)	
Median number of months in theater	5	6	0.94

* *n* = 169 for Cases group.

**Table 2:** Percentage *TGF* (rs1800469), *IL6R* (rs8192284) and *TLR4* (rs4986791) genotype frequencies in Caucasian GWI cases and controls. *p*-values were obtained by Chi-square tests comparing the two groups.

Genotype	Cases (%)	Controls (%)	Chi-Square Value	*p*
*TGF*			9.5	0.009
C/C	41.8	70.6		
C/T	45.9	23.5		
T/T	12.3	5.9		
*IL6R*			10.3	0.006
A/A	36.1	64.7		
A/C	50.9	23.5		
C/C	13.0	11.8		
*TLR4*			7.63	0.006
C/C	88.8	70.6		
C/T	11.2	29.4		
T/T	0	0		
